# A middle lobe sparing sleeve resection versus bilobectomy for right lower central non-small cell lung cancer: a retrospective propensity score matched cohort study

**DOI:** 10.1186/s13019-024-02744-5

**Published:** 2024-04-16

**Authors:** Jiongjie Wang, Jichao Guo, Haizhan Shi, Xiangru Chen, Wenxin He, Zhixin Li

**Affiliations:** 1 Department of Thoracic Surgery, Xuzhou Central Hospital, Xuzhou, 221000 China; 2 Department of Thoracic Surgery, Lanshan District People’s Hospital of Linyi City, Linyi, 276000 China; 3 Department of Thoracic Surgery, The Third Clinical Medical College of Fujian Medical University, The First Hospital of Putian, Putian, 351100 China; 4 Department of Thoracic Surgery, Shanghai Pulmonary Hospital, School of Medicine, Tongji University, No. 507 Zhengmin Road, Shanghai, 200433 China

**Keywords:** Non-small cell lung cancer, Sleeve resection, Bi-lobectomy, Residual space cavity, Right lower lobe

## Abstract

**Objectives:**

The right lower sleeve lobectomy is a rarely performed major lung resection.This study aims to evaluate the safety and effectiveness of this procedure by comparing to right lower bilobectomy in non-small cell lung cancer patients.

**Methods:**

We retrospectively reviewed a prospective database of non-small cell lung cancer patients who underwent right lower sleeve lobectomy (group S) or right lower bilobectomy (group B) from January 2014 to January 2020 in Shanghai Pulmonary Hospital. Propensity score matching method was applied to balance confounders between the two groups, resulting in 41 matched pairs.The analysis was performed to compare perioperative outcomes, long-term survival, and postoperative pulmonary volume between the two groups.

**Results:**

No significant differences in the characteristics were observed between the two matched groups.Major postoperative complications developed in 31.7% of the patients in group B and 12.1% of the patients in group S (*P* = 0.032).Intervention rate for surgical residual cavity in group B is significantly higher than those patients in group S(21.9%vs7.3%,*p* = 0.037).The postoperative right lateral and overall lung volume in group S were both significantly larger than that in group B (*P* = 0.026,*P* = 0.001,respectively).

**Conclusions:**

Compared to bi-lobectomy, a middle lobe sparing sleeve resection obtains a less prevalence of major complications, smaller postoperative residual air space and similar long-term survival for selected central right lower NSCLC patients.

**Supplementary Information:**

The online version contains supplementary material available at 10.1186/s13019-024-02744-5.

## Introduction

The advantage of sleeve lobectomy over pneumonectomy in terms of surgical and oncological outcomes in centrally located tumors has been well established [1–4]. Nowadays, sleeve lobectomy has been used in patients who can tolerate the pneumonectomy or not. The application of sleeve procedures still varies worldwide among different institutions and thoracic surgeons, especially in some complex anastomosis. Most frequently performed procedures were upper sleeve resection, right sleeve lower bi-lobectomies, and left lower sleeve lobectomies.However, reimplantation of the right middle lobe (RML) bronchus to the intermediate (IM) bronchus in right lower sleeve resection is an infrequent procedure due to a obvious mismatch in caliber of the two bronchus.For those patients whose tumor located in the right lower bronchus without invading to the IM or right middle lobe, a traditional right lower bilobectomy without peservation of middle lobe was inclined to be the first choice by most surgeons.Several reports had indicated a feasibility of right lower sleeve resections (RLS) [5–9]. However, all reports were presented with a very limited number of cases without referring to advantages or disadvantages of this middle lobe preserving procedures.The purpose of this study was to explore the surgical indication of this particular sleeve procedure and to assess its safety and efficacy in the perioperative and oncological outcomes by comparing to right lower bilobectomy(RLB).

## Patients and methods

### Patient population

Between January 2014 and January 2020, patients who underwent right lower sleeve lobectomy or right lower bilobectomy for right lower central non-small cell lung cancer (NSCLC) were enrolled in the study.Patients whose tumor invading the middle lobe bronchus or the oblique fissure were excluded.Patients with confirmed N2 or N3 lymph node metastasis by mediastinoscopy or positron emission tomography-computed tomography(PET-CT) before surgical resection were also excluded from this study.Those patients who received additional resection procedures(such as resections of thoracic walls or great vessels) were excluded as well. The clinical data including demographic characteristics, surgical procedure, tumor-node-metastasis (TNM) stage and postoperative complications were reviewed and collected.Tumor stages were adjusted to be in accordance with the eighth edition of the American Joint Commission for Cancer TNM classification.This study was approved by the review board of Shanghai Pulmonary Hospital (NO.K22-209)and the requirement for informed consent for the use of patients’ medical record was waived.All methods were performed in accordance with the Declaration of Helsinki.

### Preoperative assessment

All patients underwent a battery of examinations before the operation, including chest computed tomography (CT), abdominal ultrasound, brain magnetic resonance imaging, bone emission CT, spirometry, and fiberoptic bronchoscopy.18 F-fluorodeoxyglucose positron emission tomography (PET) was performed in cases with suspicious metastasis. Fluorescent bronchoscopy was conducted to reveal the location of the tumor and discern the possibly involved areas of bronchial mucosa.Cervical mediastinoscopy with biopsy of lymph node stations 2, 4 (both left and right), and 7 was performed to exclude N2/N3 disease detected by CT scan.According to the findings of chest CT and bronchoscopy, a preliminary surgical plan was made and the feasibility of the RLS procedure was evaluated by the same surgical team preoperatively.

### Surgical technique

General anesthesia was administered to all patients with a double lumen intubation. In patients who underwent thoracotomy, a standard posterolateral incision of 20–30 cm was performed in the 4th or 5th intercostal space. In patients who underwent uniportal video-assisted thoracic surgery (VATS), a 4 cm incision was made at the anterior axillary line in the fourth intercostal space.After complete exposure of the fissure, pulmonary artery, bronchus, and vein, technical feasibility of a RLS was reassessed.If possible, the proximal of intermediary bronchus was cut transversely and the orifice of middle lobe was resected obliquely, respectively.A frozen section of bronchial margin and N1 lymph node were routinely checked under pathological examination.When a negative margin was confirmed and N1 lymph node metastasis was ruled out, the anastomosis between intermediate bronchus and middle lobe bronchus was completed by continuous suture with a single 3 − 0 Prolene (Ethicon, Inc, Somerville, NJ).Any discrepancy in size of the orifice between the intermediate bronchus and the middle lobe bronchus was corrected by adjusting the membranous part of the intermediate bronchus in the continuous suture(Video 1 showed an uni-port VATS right lower sleeve resection).While in bilobectomy group, the intermediate bronchus was transected and closed with a stapler(Ethicon, Inc, ECR45B). Air leakage were checked by a sustained airway pressure of 30 cm H_2_O. Radical lymph nodes dissections were also routinely performed in both groups.Bronchoscopy was conducted for sputum clearness or anastomotic examination when there was a suspected atelectasis or bronchial stenosis/fistula.

### Outcomes assessment

Perioperative outcomes included duration of operation, blood loss, surgical margins, tube length of drainage, postoperative complications, perioperative mortality (defined as any death occurring within 30 days after operation or any death during hospitalization), and readmission rate.In this study, all complications were graded by the Clavien-Dindo classification system, while major complications were defined as grade II or above.Intervention for surgical residual cavity is defined as additional thoracic drainage due to a large surgical residual cavity or severe pulmonary atelectasis caused by pleural effusion after initial removal of the drainage tube.Every patient underwent a CT-scan at an end of inspiration 3 months after surgey.A Mimics Medical 3-D 20.0 software (Materialise, Belgium) was used for the construction and evaluation of postoperative pulmonary residual volume.

Overall survival(OS), and disease free survival(DFS) were defined as the time from surgery to death and recurrence, respectively. The follow-up data were obtained from telephone calls, letters, or direct outpatient examinations from the day of surgery.All patients underwent follow-up at 3-month intervals for the first year, 6-month intervals for the second year, and yearly thereafter to monitor disease progression and evaluate the appearance of bronchial flaps with chest CT and fiberoptic bronchoscopy.

### Statistical analysis

Statistical analyses were performed using SPSS 22.0 (SPSS, Inc, Chicago, Ill). Continuous variables were presented as means ± standard deviation or medians (range), and categorical variables were presented as counts or rates. A chi squared test or Fisher´s exact test was used to compare dichotomous variables. Survival analysis was performed using the Kaplan-Meier curves and compared by log-rank test. Propensity score matching was used to mitigate discrepancies in the characteristics of the study cohort that could influence our outcomes. Cases were matched 1:1 with a caliper size of 0.01.Variables used for matching were age, FEV1,FEV1%, gender, and surgical procedure.A *P*-value less than 0.05 was considered statistically significant.

## Results

**Fig. 1:**
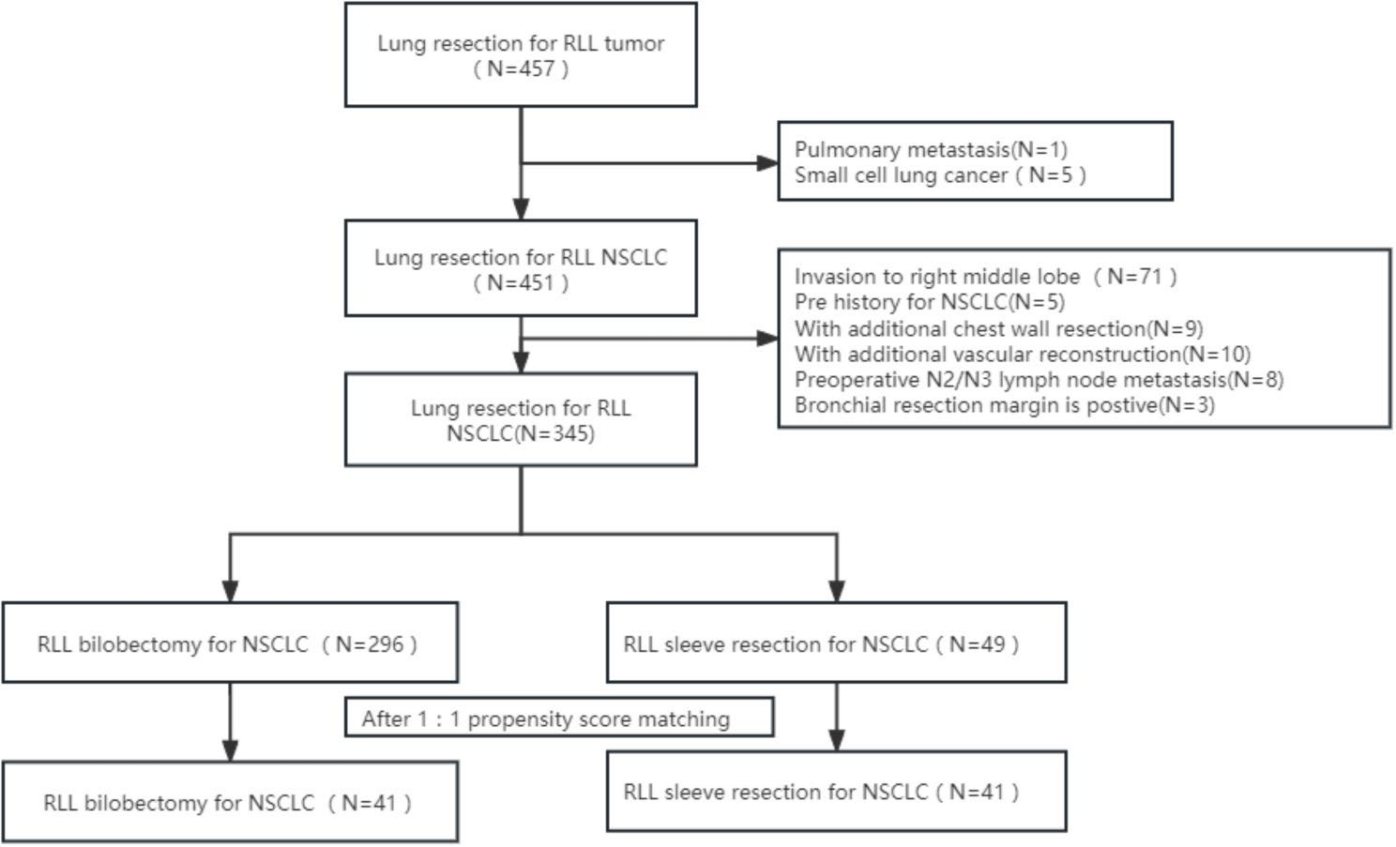
Flow chart for inclusion and exclusion of patients

A total of 451 patients with right lower central NSCLCs underwent major lung resections in our department.There were 296 RLBs and 49 RLSs were finally included to our study. After calculating the propensity scores (ratio = 1:1), 41 pairs were matched. (Fig. 1)

### Unmatched population

Detailed characteristics of the patients are listed in Table 1. The basic line of patients who undergoing bilobectomy were quite similar compared to those in the sleeve resection group except for the lung function, CCI score and cTNM stage before PSM matching.The FEV1(2.35 ± 0.53 vs.2.53 ± 0.67 L, *P* = 0.049) and FEV1%(90.21 ± 17.90 vs.78.11 ± 12.75%,*P* = 0.000)of lung function in group B was much better compared with group S; The CCI score(>2) of patients in group B is significantly less than those patients in group S.No significant differences were found between the VATS and open groups with regard to age at surgery, gender, smoking history, neoadjuvant therapy, surgical procedure, preoperative lung volume and tumor pathology.

### Matched population

The propensity score matching (PSM) identified 41 cases in each group, baseline characteristics of the matched patients were listed in Table 1.No significant differences were found in all variables of demographic characteristics between the two groups after PSM.The R0 resection rate in both groups was 100%.In group S, among 41 patients who underwent this bronchial end-to-end anastomosis procedure.The pathologic examinations disclosed 30 squamous cell carcinomas,5 adenocarcinomas,1 adenosquamous carcinoma,1 adenoid cystic carcinoma,1 large cell carcinoma, and 3 lung carcinoid carcinomas.The lymph node involvements were classified as N0 in 33 patients, N1 in 4 patients, and N2 in 6 patients. According to the 8th TNM classification, 20 were in stage Ib, 8 were in stage IIa, 6 in stage IIb, 5 in stage IIIa, and 2 in stage IIIb. In group B, The pathologic examinations disclosed 30 squamous cell carcinomas, 6 adenocarcinomas,1 adenoid cystic carcinoma, 1 large cell carcinoma, and 3 lung carcinoid carcinomas.Based on the 8th TNM staging system for NSCLC, the distribution of pathologic stages of 41 patients in group B were stage I, stage II, and stag III were 48.8%,31.7%,19.5%, respectively.

**Table 1:** Demographics and Clinical Data before PSM and after PSM

	Before PSM			After PSM		
Variables	Group B N (%)n = 296	Group S N (%)n = 49	*P*	Group B N (%)n = 41	Group S N (%)n = 41	*P*
**Gender**			0.090			0.305
Male	228(77.0%)	43(87.7%)		40	38	
Female	68(23.0%)	6(12.3%)		1	3	
**Age**	61.0 ± 9.7	61.4 ± 8.01	0.802	61.9 ± 9.0	61.6 ± 7.9	0.867
**Smoking**			0.760			1.000
Yes	160(54.1%)	28(57.1%)		19	19	
No	136(45.9%)	21(42.9%)		22	22	
**Neotherapy**			0.615			0.305
Yes	24(8.1%)	3(6.1%)		1	3	
No	272(91.9%)	46(93.9%)		40	38	
**FEV1**	2.35 ± 0.53	2.53 ± 0.67	0.049	2.49 ± 0.56	2.57 ± 0.68	0.604
**FEV1%**	90.21 ± 17.90	78.11 ± 12.75	0.000	76.27 ± 6.85	76.74 ± 13.45	0.853
**CCI score**			0.018			0.693
≤2	280(94.6%)	45(91.8%)		37(90.2%)	38(92.7%)	
>2	16(5.4%)	4(8.2%)		4(9.8%)	3(7.3%)	
**Surgical procedure**			0.589			0.814
Open/VATS-assisted	128(43.2%)	19(38.8%)		13	14	
VATS	168(56.8%)	30(61.2%)		28	27	
**Pre right lung volume**	2.66 ± 0.27	2.25 ± 0.75	0.271	2.58 ± 0.30	2.54 ± 0.59	0.875
**Pre overall lung volume**	5.07 ± 0.48	4.23 ± 0.14	0.227	4.99 ± 0.47	4.71 ± 0.12	0.597
**Tumor pathology**			0.190			0.913
**Squamous cell carcinoma**	208	35		30	30	
**Adenocarcinoma**	60	6		6	5	
**Other non-small cell carcinoma**	28	8		5	6	
**cTNM stage**			0.001			0.949
**Ib**	116	24		20	20	
**II(a-b)**	168	17		13	14	
**III(a-b)**	12	8		8	7	

### Perioperative outcomes before and after PSM matching

Before PSM matching, the overall 30-day mortality of group B and group S was 2.7%(8/296) and 2.1%(1/49),respectively, which is no significantly different(*P* = 0.788).However, there was a significant difference in major postoperative complications between group B and group S(36.7% vs. 27.3%,*P* = 0.044) before PSM matching.After PSM matching, a 30-day mortality of group S is 2.4%(1/41).Major postoperative complications occurred in 5 (12.1%) patients: bronchopleural fistula (1/41), pulmonary atelectasis and infection (1/41), hemothorax (1/41), chylothorax (1/41), and prolonged air leak (1/41).While in group B, the overall 30-day mortality and morbidity rates were 2.4% and 31.7%, respectively. Major complications developed in 13 patients in group B during the postoperative period (bronchopleural fistula in 1 patients, pulmonary embolism in 1 patients, prolonged air leak in 5 patients, pneumonia or atelectasis in 4, in 2, hemothorax in 1, and chylothorax in 1).The mean operation time was 163.5 ± 54.9 min in group B and 171.1 ± 60.8 min in group S (*P* = 0.464).The mean postoperative tube length in group B is longer than that in group S (12.5 ± 2.9days vs9.1 ± 2.7days,*P* = 0.063),but without a statistical differences.Intervention rate for Surgical Residual Cavity in group B is significantly higher than those patients in group S(21.9%vs7.3%,*p* = 0.037).The pre/post thoracic cavity volume ratio after the interval of 3 months postoperatively between the two groups indicated a significant difference (*P* = 0.001).(see Table 2).

**Table 2:** Perioperative outcome between the two matched groups

Variables	Group B N (%) N = 41	Group S N (%) N = 41	P-value
Operation time (min)	163.5 ± 54.9	171.1 ± 60.8	0.464
Blood loss(mL)	100(50-1100)	100(30–450)	0.502
Blood transfusion(No/Yes)	39/2	41/0	0.152
Mortality	1(2.4%)	1(2.4%)	1.000
Postoperative complication	13(31.7%)	5(12.1%)	0.032
Bronchopleural fistula(BPF)	1(2.4%)	1(2.4%)	
Pulmonary embolism	1(2.4%)	0(0.0%)	
Pulmonary infection	4(9.8%)	1(2.4%)	
Air leak	5(12.2%)	1(2.4%)	
Chylothorax	1(2.4%)	1(2.4%)	
Hemothorax	1(2.4%)	1(2.4%)	
Indwelling tube stay(day)	12.5 ± 2.9	9.1 ± 2.7	0.063
Intervention for Surgical Residual Cavity	9(21.9%)	3(7.3%)	0.037
Post right lung volume(L)	1.19 ± 0.26	1.56 ± 0.22	0.026
Pre/post lung volume ratio	1.405 ± 0.258	1.201 ± 0.140	0.001

### Long-term outcome before and after PSM matching

**Fig. 2:**
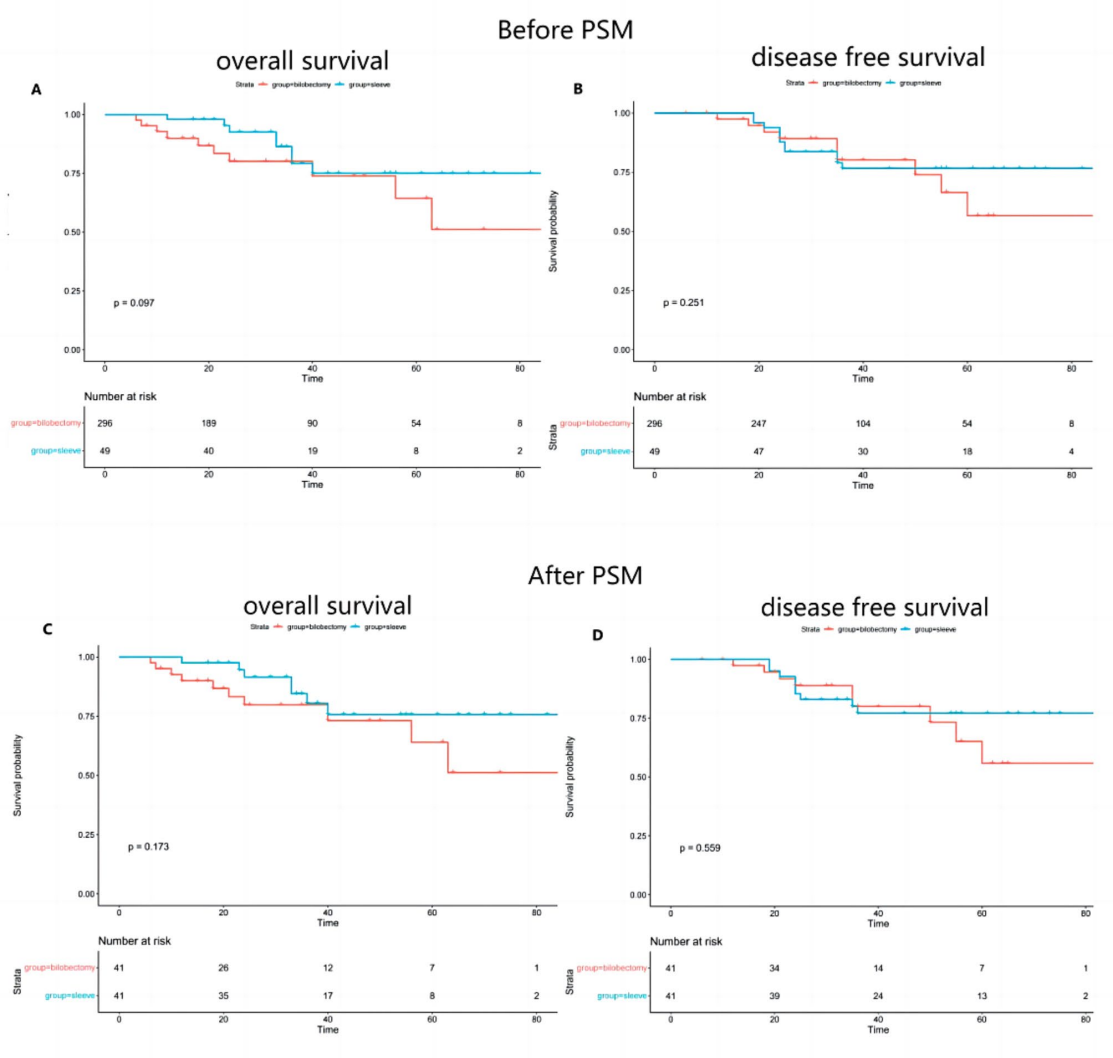
**(A)** The overall survival in the bi-lobectomy group and sleeve resection group before PSM matching; (**B)** The disease-free survival in the bi-lobectomy group and sleeve resection group before PSM matching. (**C)** The overall survival in the bi-lobectomy group and sleeve resection group after PSM matching; (**D)** The disease-free survival in the bi-lobectomy group and sleeve resection group after PSM matching

The median follow-up period after surgery was 46.8 months in the group B and 47.8 months in group S before PSM matching.There were no significant differences in 5-year OS(75.0% vs. 64.4%,*P* = 0.097) and DFS(76.6% vs. 56.6%,*P* = 0.251) between group S and group B before PSM matching(Fig. 2, A and B).In the matched cohort, the median follow-up period was 47.6 months and 48.3 months in the group B and group S, respectively. There were no significant differences in 5-year OS(75.8% vs. 64.0%,*P* = 0.173) and DFS(77.1% vs. 55.9%,*P* = 0.559) between group S and group B after PSM matching (all *P* > 0.05) (Fig. 2, C and D).

## Disscussion

**Fig. 3:**
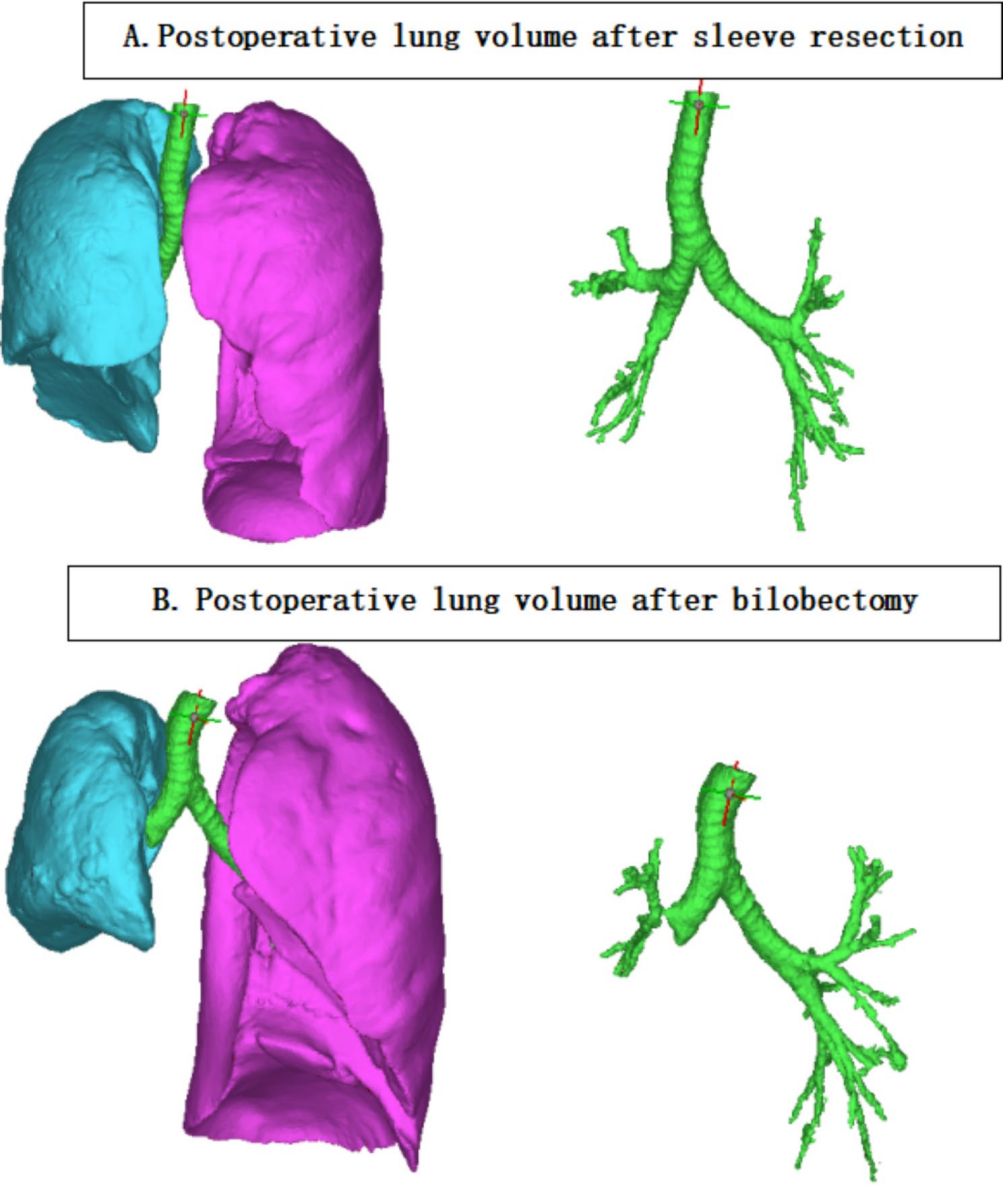
**(A)** postoperative 3-D construction of bronchus and volume after right lower sleeve resection; (**B)** postoperative 3-D construction of bronchus and volume after right lower bilobectomy

For a central tumor located in the lower lobe bronchus without invading IM bronchus and middle lobe, the majority of surgeons believe that the ML offers little contribution to lung function and a bilobectomy was perfered to a more complicated RLSThere are some unavoidable challenges in right lower sleeve lobectomy.Firstly, it is difficult to perform an end-to-end anastomosis due to a mismatch in caliber between middle lobe bronchus and the intermediate bronchus.Secondly, there was a poor exposure for bronchial anastomosis especially behind the middle lobe artery.Although it is true that sleeve lobectomy is technically more demanding than bilobectomy, sleeve resection has gradually been an attractive procedure chosen by experienced thoracic surgeons for lung cancer patients due to progress of technology and equipment.Besides, bilobectomy, especially lower bilobectomy incurs high morbidity and mortality [10, 11].It seems that RLS with preservation of the middle lobe may be an alternative for right lower centrally located tumors.When a complete oncological resection could be obtained, theoretically a reserved pulmonary parenchyma may result in a better postoperative lung function and quality of life of the patient.But there is still a lack of evidence of better short and long-term outcomes followed by RLSs.Therefore, we retrospectively evaluated this particular procedure by comparing the short- and long-outcomes of RLSs with RLBs in central NSCLC.To obtain a more credible comparison, a PSM method was applied in this study to balance key variables and mitigate the selection bias between the two groups (See Fig. 3).

This study indicate that RLS could be a safe and effective technique in spite of complicated surgical procedure.The 30-day mortality after sleeve lobectomy ranges from 2.14 to 12.6% and is reported at about 1.4-4.3% for bilobectomy in high volume thoracic surgery centers [12–15].Our findings are largely consistent with the data reported in the literature.Mortality between the two groups in our study is similar before and after PSM, which indicate that a more complex RLS is safe without increasing perioperative death rate compared to a RLB. Besides, a low incidence of BPF(2.4%) is acceptable for group S, indicating that complex anastomosis and reconstruction in bronchial sleeve resection does not increase BPF rate.The novel method to correct caliber disparity by cutting the orifice of middle lobe obliquely in our center may contribute to the relatively low rate of BPF in the RLSs group.

A benefit of perioperative outcomes in preservation of middle lobe had been reported by a series of studies.Kocaturk et al. reported a shorter hospitalization time and less postoperative complication for a right sleeve lower lobectomy compared to a bilobectomy [9].Ludwig et al. also reported a lower incidence of complication and a benefit of postoperative lung function improvement by comparing 21 sleeve lobectomies and 15 bilobectomies [16].Our study also indicated a lower incidence of postoperative complications after RLSs compared to RLBs(12.1%vs 31.7%,*P* = 0.032).Patients after RLBs suffered more pulmonary infection and air leak; Moreover, intervention for residule pleural space was more frequently required in RLBs group as well.Larger postoperative residual spaces in chest cavity always correlates with higher risk of pulmonary air leak and unsorbed effusion, which required redrainage or reoperation. Thomas et al. reported a rate of pleural space complications can be as high as 16.2% in a cohort of 1831 bilobectomies and more frequently in lower bilobectomies than upper bilobectomies [10]. But there is still lack of evidence for the hypothesis that a bilobectomy is more inclined to form a residual air space and incur more pleural space complication than a sleeve lobectomy.Different from an subjective judgment of residual space by chest x-rays or CT scan, our study validated this difference of residual cavity volume in a quantitative method by a 3-D construction of postoperative CT scan. Fewer incidences of prolonged air leak and lower rate of intervention for residual cavity in group S, in particular, can be explained by a smaller residual space from our data.Besides, we hope a prospective clinical trail by combining this 3-D quantitative method with lung function tests might validate the benefit of lung function of RLSs in the future.

A survival benefit of parenchymal preservation between sleeve resection and pneumonectomy has been widely validated [17, 18].Compared to pneumonectomy, a lung tissue sparing sleeve resection obtains a lower incidence of local recurrence and better oncological outcome.Similarly, no significant difference in postoperative recurrence rate was observed and a comparable overall survival and disease-free survival were found between RLS and RLB groups in our study.A 100% R0 rate was achieved in all right lower sleeve resections, which confirming the oncological efficacy of this middle lobe sparing procedure.What is worth mentioning, the overall survival in sleeve resection group showed a trend towards superiority than the bilobectomy group after 36 months, which might be interpreted by that patients receiving bilobectomy were associated with a higher non-cancer death risk owning to a limited cardiopulmonary reservation.

The choice to perform one surgical procedure was mainly depended on its efficacy and feasibility.Based on our results, the parenchyma-sparing procedure could be as alternative approach to bilobectomy for central NSCLC when it was technically possible. Meanwhile, what we should not ignore was the impact of the learning curve inherent to this complex procedure.Achieving a proper anastomosis is technically difficult in RLSs because of the differences in bronchial caliber and the anastomotic tension is much greater than in other sleeve lobectomies.In our series, the orifice of middle lobe was cut obliquely and the bronchial anastomosis tension was declined by releasing the anterior hilar structure around the middle lobe.With the development of surgical skills and devices, VATS technique has been widely applied in major pulmonary resection [19].As an experienced thoracic center in China, our study reported a more than 50% paitents(56.8%) underwent bilobectomies and sleeve resections(61.2%) by VATS approaches without increased mortality and morbidity.However, for surgeons with inadequate surgical proficiency, we advocated that more rigorous preoperative evaluation and more cautious intraoperative judgements are needed before decision makings to guarantee the safety of this challenging procedure.

### Limitations

The limitations of our study are obvious.There was an inevitable selection bias due to a retrospective nature and a relatively limited number of cases; Besides, the operations were performed by different surgeons in our study; Moreover, general survival was calculated rather than survival by stages due to a small number of patients. Theoretically, right lower sleeve lobectomy preserves more pulmonary parenchyma and so better expansion of lung tissue which may lead to a better respiratory function. Unfortunately, the patients’ postoperative lung function was not compared between the groups due to missing data.A prospective clinical trail in the future is required to validate a possible benefit of lung function of RLSs.

## Conclusion

Based on our results, a right lower sleeve lobectomy is associated with lower incidence of postoperative complications, comparable oncological outcome and smaller postoperative residual air spaces, which can be an appropriate alternative to right lower bilobectomy in selected patients at experienced centers.

### Electronic supplementary material

Below is the link to the electronic supplementary material.

Supplementary Material 1

## Data Availability

No datasets were generated or analysed during the current study.

## Abbreviations

NSCLC: Non-Small Cell Lung Cancer
VATS: Video-Assisted Thoracic Surgery
RML: Right Middle Lobe
RLL: Right Lower Lobe
CT: Computed Tomography
PET: Positron Emission Tomography
RLS: Right Lower Lobe Sleeve Resections
RLB: Right Lower Bi-lobectomy
OS: Overall Survival
DFS: Disease-Free Survival
BPF: Bronchopleural Fistula
PSM: Propensity Score Matching

## References

[1] Deslauriers J, Grégoire J, Jacques LF (2004). Sleeve lobectomy versus pneumonectomy for lung cancer: a comparative analysis of survival and sites or recurrences. Ann Thorac Surg.

[2] Maurizi G, Ciccone AM, Rendina EA (2019). The advantage of sleeve lobectomy over pneumonectomy. J Thorac Disease.

[3] Predina JD, Kunkala M, Aliperti LA (2010). Sleeve lobectomy: current indications and future directions. Ann Thorac Cardiovasc Surg.

[4] Chen Y, Zhang L, Yan B (2020). Feasibility of sleeve lobectomy after neo-adjuvant chemo-immunotherapy in non-small cell lung cancer. Translational Lung Cancer Res.

[5] Boudaya MS, Abid W, Mlika M (2016). Sleeve right lower lobectomy: a rarely performed extended resection. Indian J Surg.

[6] Ohata K, Zhang J, Ito S (2013). Right lower lobe sleeve resection: bronchial flap to correct caliber disparity. Ann Thorac Surg.

[7] Tanaka K, Nakajima T, Morimoto J, Yoshino I (2015). Right lower sleeve lobectomy with double-barreled bronchoplasty for a centrally located lung cancer. Ann Thorac Surg.

[8] Hamasaki H, Shirakami C, Yamada T (2021). Specific techniques for right sleeve lower lobectomy: four case reports. Surg Case Rep.

[9] Kocaturk CI, Saydam O, Sezen CB (2020). Is right sleeve lower lobectomy necessary? Is it safe?. Thorac Cardiovasc Surg.

[10] Thomas PA, Falcoz PE, Bernard A (2016). Bilobectomy for lung cancer: contemporary national early morbidity and mortality outcomes. Eur J Cardiothorac Surg.

[11] Xie D, Deschamps C, Shen RK (2015). Bilobectomy Versus Lobectomy for Non-small Cell Lung Cancer: a comparative study of outcomes, long-term survival, and Quality of Life. Ann Thorac Surg.

[12] Kim AW, Faber LP, Warren WH (2010). Bilobectomy for non-small cell lung cancer: a search for clinical factors that may affect perioperative morbidity and long-term survival. J Thorac Cardiovasc Surg.

[13] Galetta D, Solli P, Borri A (2012). Bilobectomy for lung cancer: analysis of indications, postoperative results, and long-term outcomes. Ann Thorac Surg.

[14] Tagawa T, Iwata T, Nakajima T (2016). Evolution of a lung-sparing strategy with sleeve lobectomy and induction therapy for non-small cell Lung Cancer: 20-Year experience at a single Institution. World J Surg.

[15] Yildizeli B, Fadel E, Mussot S (2007). Morbidity, mortality, and long-term survival after sleeve lobectomy for non-small cell lung cancer. Eur J Cardiothorac Surg.

[16] Ludwig C, Morand P, Schnell J, Stoelben E (2009). Preserving middle lobe to improve lung function in non-small-cell lung cancer. Asian Cardiovasc Thorac Ann.

[17] Chen J, Soultanis KM, Sun F (2021). Outcomes of sleeve lobectomy versus pneumonectomy: a propensity score-matched study. J Thorac Cardiovasc Surg.

[18] Maurizi G, Ciccone AM, Rendina EA (2019). The advantage of sleeve lobectomy over pneumonectomy. J Thorac Dis.

[19] Gao HJ, Jiang ZH, Gong L (2019). Video-assisted vs Thoracotomy Sleeve Lobectomy for Lung Cancer: a propensity matched analysis. Ann Thorac Surg.

